# *N*,*N*′,*N*′′-Triphenyl­guanidinium 5-nitro-2,4-dioxo-1,2,3,4-tetra­hydro­pyrimidin-1-ide

**DOI:** 10.1107/S1600536808014244

**Published:** 2008-05-17

**Authors:** P. S. Pereira Silva, S. R. Domingos, M. Ramos Silva, J. A. Paixão, A. Matos Beja

**Affiliations:** a CEMDRX, Physics Department, University of Coimbra, P-3004-516 Coimbra, Portugal

## Abstract

In the title compound, C_19_H_18_N_3_^+.^C_4_H_2_N_3_O_4_^−^, the dihedral angles between the phenyl rings and the plane defined by the central guanidinium fragment are in the range 41.3 (1)–66.6 (1)°. The pyrimidine ring of the anion is distorted towards a boat conformation and the nitro group is rotated 11.4 (2)° out of the uracil plane. Hydrogen bonds assemble the ions in infinite helical chains along the *b* axis.

## Related literature

For the non-linear optical properties of 5-nitro­uracil, see: Puccetti *et al.* (1993 ▶), Youping *et al.* (1992 ▶). For reports of other triphenyl­guanidine salts, see: Pereira Silva *et al.* (2006 ▶, 2007*a* ▶,*b* ▶), Pereira Silva, Cardoso *et al.* (2007 ▶). For related literature, see: Allen *et al.* (1987 ▶); Kemme *et al.* (1988 ▶); Klement *et al.* (1995 ▶); Largent *et al.* (1987 ▶); Pettier & Byrn (1982 ▶); Rao *et al.* (1995 ▶); Weber *et al.* (1986 ▶); Zyss *et al.* (1993 ▶).
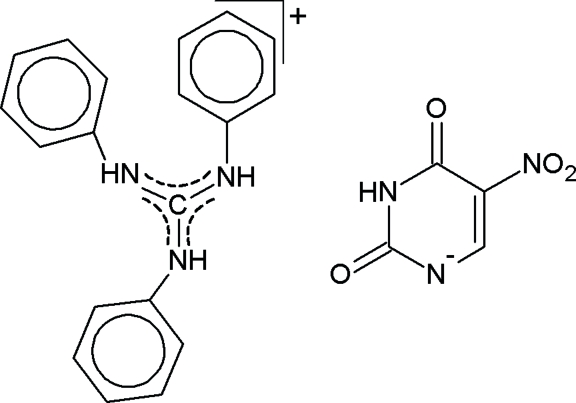


## Experimental

### 

#### Crystal data

C_19_H_18_N_3_^+^·C_4_H_2_N_3_O_4_^−^
*M* *_r_* = 444.45
Monoclinic, 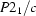

*a* = 10.7495 (4) Å
*b* = 15.6892 (7) Å
*c* = 15.5624 (7) Å
β = 123.456 (3)°
*V* = 2189.74 (18) Å^3^
*Z* = 4
Mo *K*α radiation
μ = 0.10 mm^−1^
*T* = 293 (2) K
0.34 × 0.20 × 0.12 mm

#### Data collection

Bruker APEX2 CCD area-detector diffractometer
Absorption correction: multi-scan (*SADABS*; Sheldrick, 2003 ▶) *T*_min_ = 0.822, *T*_max_ = 0.989
47807 measured reflections
5534 independent reflections
2650 reflections with *I* > 2σ(*I*)
*R*_int_ = 0.086

#### Refinement

*R*[*F* ^2^ > 2σ(*F* ^2^)] = 0.050
*wR*(*F* ^2^) = 0.154
*S* = 0.99
5534 reflections
299 parameters
H-atom parameters constrained
Δρ_max_ = 0.22 e Å^−3^
Δρ_min_ = −0.17 e Å^−3^

### 

Data collection: *APEX2* (Bruker, 2005 ▶); cell refinement: *SAINT* (Bruker, 2005 ▶); data reduction: *SAINT*; program(s) used to solve structure: *SHELXS97* (Sheldrick, 2008 ▶); program(s) used to refine structure: *SHELXL97* (Sheldrick, 2008 ▶); molecular graphics: *PLATON* (Spek, 2003 ▶); software used to prepare material for publication: *SHELXL97*.

## Supplementary Material

Crystal structure: contains datablocks global, I. DOI: 10.1107/S1600536808014244/bt2707sup1.cif

Structure factors: contains datablocks I. DOI: 10.1107/S1600536808014244/bt2707Isup2.hkl

Additional supplementary materials:  crystallographic information; 3D view; checkCIF report

## Figures and Tables

**Table 1:** Hydrogen-bond geometry (Å, °)

*D*—H⋯*A*	*D*—H	H⋯*A*	*D*⋯*A*	*D*—H⋯*A*
N6—H6*A*⋯O2^i^	0.86	1.94	2.794 (2)	174
N7—H7⋯N1^i^	0.86	2.21	2.934 (2)	142
N8—H8⋯O4^ii^	0.86	2.05	2.887 (2)	163

Symmetry codes: (i) 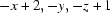; (ii) 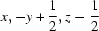.

**Fig. 1.**
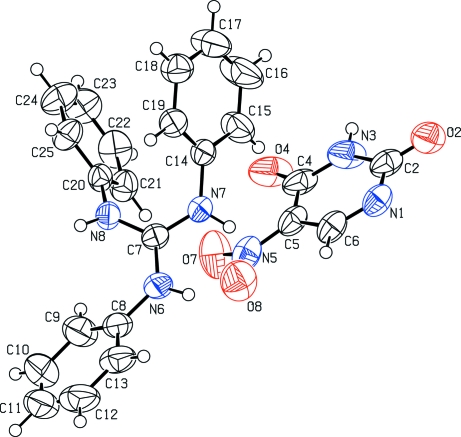
ORTEPII (Spek,2003) plot of the title compound. Displacement ellipsoids are drawn at the 50% probability level.

**Fig. 2.**
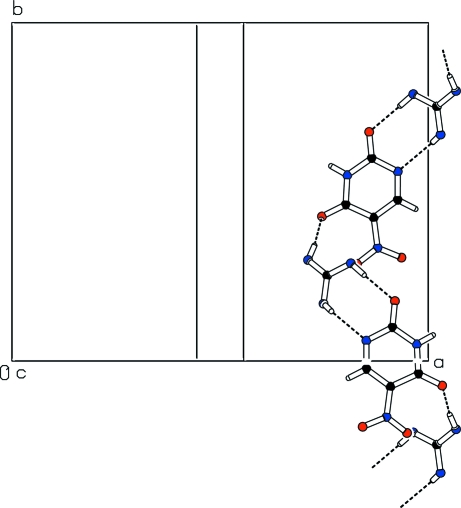
Packing diagram, viewed down the c axis, with the hydrogen bonds depicted as dashed lines. The phenyl rings have been omitted for clarity.

## supplementary crystallographic information

### Comment

5-Nitrouracil is currently of prime interest to the non-linear optical community (Puccetti *et al.*, 1993; Youping *et al.*, 1992) and is also of relevance to the biological and pharmaceutical sciences (Rao *et al.*, 1995; Pettier & Byrn, 1982).

Much of the interest in guanidine compounds and its derivatives is due to their biological activity, in particular their neuroleptic and antipsychotic properties (Weber *et al.*, 1986; Largent *et al.*, 1987). Our interest is focused on the physical properties of guanidine compounds, which are regarded as potentially interesting for non-linear optics applications (Zyss *et al.*, 1993). We are currently engaged in a research project aimed at investigating the structural, dielectric and optical properties of triphenylguanidine compounds.

Compound (I) (Fig.1) is built up from triphenylguanidinium cations and 5-nitrouracilate anions. The pyrimidine ring is almost planar with a slight distortion towards a boat configuration. The nitro group is rotated 11.4 (2)° out of the plane of the uracil fragment. The central guanidine fragment of the cation of the title salt is planar with bond lengths and angles close to those expected for a central C*sp*^2^ atom, accounting for some charge delocalization between the three C—N bonds. The bond lengths C7—N6 [1.333 (2) Å], C7—N7 [1.330 (2) Å] and C7—N8 [1.337 (2) Å] are comparable with literature averages for substituted and unsubstituted guanidinium cations (1.321 and 1.328 Å, respectively; Allen *et al.*, 1987)

The dihedral angles between the ring planes and the plane defined by the central guanidinium fragment are 41.3 (1)(C8—C13), 57.5 (1)(C14—C19) and 66.6 (1)° (C20—C25). The corresponding angles for other triphenylguanidinium salts reported in the literature are within the range 32.6 (3)–70.2 (3)° (Kemme *et al.*, 1988; Klement *et al.*, 1995; Pereira Silva *et al.*, 2006, 2007*a*, 2007*b*, Pereira Silva, Cardoso *et al.*,(2007).

The anions and cations are linked into infinite helical chains running parallel to the *b* axis, *via* hydrogen bonds involving all the NH groups of the guanidinium fragment, the carbonyl O atoms and the deprotonated N atom of the anion (Fig. 2, Table 2). Atoms O2 and N1 accept each one H atom across a crystallographic centre of symmetry, while the O4 atom accept one hydrogen from the N8 atom related by a twofold screw axis.

### Experimental

The title compound was prepared by adding 5-nitrouracil (Aldrich, 98%, 1 mmol) to triphenylguanidine (TCI 97%, 1 mmol) in a ethanol solution (80 ml). The solution was slowly warmed and then left to evaporate under ambient conditions. After a few days, small yellow transparent single crystals were deposited.

### Refinement

All H atoms were located in a difference Fourier synthesis, placed at calculated positions and refined as riding on their parent atoms, using *SHELXL97* (Sheldrick, 2008) defaults [C—H = 0.93 Å, N—H = 0.86 Å and *U*_iso_(H) = 1.2*U*_eq_(C,*N*)].

### Crystal data

**Table:**

C_19_H_18_N_3_^+^·C_4_H_2_N_3_O_4_^–^	*F*_000_ = 928
*M**_r_* = 444.45	*D*_x_ = 1.348 Mg m^−^^3^
Monoclinic, *P*2_1_/*c*	Mo *K*α radiation λ = 0.71073 Å
Hall symbol: -P 2ybc	Cell parameters from 5110 reflections
*a* = 10.7495 (4) Å	θ = 2.3–21.6º
*b* = 15.6892 (7) Å	µ = 0.10 mm^−^^1^
*c* = 15.5624 (7) Å	*T* = 293 (2) K
β = 123.456 (3)º	Block, yellow
*V* = 2189.74 (18) Å^3^	0.34 × 0.20 × 0.12 mm
*Z* = 4	

### Data collection

**Table:**

Bruker APEX2 CCD area-detector diffractometer	5534 independent reflections
Radiation source: fine-focus sealed tube	2650 reflections with *I* > 2σ(*I*)
Monochromator: graphite	*R*_int_ = 0.086
*T* = 293(2) K	θ_max_ = 28.6º
φ and ω scans	θ_min_ = 2.0º
Absorption correction: multi-scan(SADABS; Sheldrick, 2003)	*h* = −14→14
*T*_min_ = 0.822, *T*_max_ = 0.989	*k* = −20→21
47807 measured reflections	*l* = −20→20

### Refinement

**Table:**

Refinement on *F*^2^	Hydrogen site location: inferred from neighbouring sites
Least-squares matrix: full	H-atom parameters constrained
*R*[*F*^2^ > 2σ(*F*^2^)] = 0.050	*w* = 1/[σ^2^(*F*_o_^2^) + (0.0713*P*)^2^ + 0.1061*P*] where *P* = (*F*_o_^2^ + 2*F*_c_^2^)/3
*wR*(*F*^2^) = 0.154	(Δ/σ)_max_ < 0.001
*S* = 0.99	Δρ_max_ = 0.22 e Å^−^^3^
5534 reflections	Δρ_min_ = −0.17 e Å^−^^3^
299 parameters	Extinction correction: SHELXL97 (Sheldrick, 2008), Fc^*^=kFc[1+0.001xFc^2^λ^3^/sin(2θ)]^-1/4^
Primary atom site location: structure-invariant direct methods	Extinction coefficient: 0.0068 (11)
Secondary atom site location: difference Fourier map	

### Special details

**Table:**

Geometry. All e.s.d.'s (except the e.s.d. in the dihedral angle between two l.s. planes) are estimated using the full covariance matrix. The cell e.s.d.'s are taken into account individually in the estimation of e.s.d.'s in distances, angles and torsion angles; correlations between e.s.d.'s in cell parameters are only used when they are defined by crystal symmetry. An approximate (isotropic) treatment of cell e.s.d.'s is used for estimating e.s.d.'s involving l.s. planes.
Refinement. Refinement of *F*^2^ against ALL reflections. The weighted *R*-factor *wR* and goodness of fit *S* are based on *F*^2^, conventional *R*-factors *R* are based on *F*, with *F* set to zero for negative *F*^2^. The threshold expression of *F*^2^ > σ(*F*^2^) is used only for calculating *R*-factors(gt) *etc*. and is not relevant to the choice of reflections for refinement. *R*-factors based on *F*^2^ are statistically about twice as large as those based on *F*, and *R*- factors based on ALL data will be even larger.

### Fractional atomic coordinates and isotropic or equivalent isotropic displacement parameters (Å^2^)

**Table:**

	*x*	*y*	*z*	*U*_iso_*/*U*_eq_	
O2	0.82985 (16)	−0.17695 (11)	0.60642 (13)	0.0729 (5)	
O4	0.61600 (16)	0.07278 (11)	0.59408 (15)	0.0968 (6)	
O7	0.7653 (2)	0.21285 (11)	0.59093 (15)	0.0949 (6)	
O8	0.9257 (2)	0.19450 (12)	0.55194 (16)	0.0940 (6)	
N1	0.93683 (17)	−0.06007 (12)	0.58600 (13)	0.0581 (5)	
N3	0.72353 (18)	−0.04940 (13)	0.59167 (16)	0.0759 (6)	
H3	0.6532	−0.0761	0.5911	0.091*	
N5	0.8406 (2)	0.16564 (13)	0.57455 (13)	0.0658 (5)	
C2	0.8316 (2)	−0.09943 (17)	0.59504 (16)	0.0596 (6)	
C4	0.7147 (2)	0.03782 (16)	0.58914 (17)	0.0668 (6)	
C5	0.8282 (2)	0.07584 (14)	0.57999 (14)	0.0555 (5)	
C6	0.9311 (2)	0.02398 (15)	0.57896 (15)	0.0585 (6)	
H6	1.0030	0.0506	0.5727	0.070*	
N6	0.93433 (16)	0.28871 (10)	0.33661 (12)	0.0538 (4)	
H6A	1.0086	0.2545	0.3590	0.065*	
N7	0.79767 (16)	0.16933 (10)	0.31325 (12)	0.0503 (4)	
H7	0.8742	0.1468	0.3667	0.060*	
N8	0.67626 (16)	0.29829 (10)	0.24472 (12)	0.0491 (4)	
H8	0.6759	0.3411	0.2101	0.059*	
C7	0.80155 (19)	0.25245 (12)	0.29824 (14)	0.0464 (5)	
C8	0.9677 (2)	0.37724 (13)	0.34492 (14)	0.0539 (5)	
C9	0.8938 (3)	0.43773 (15)	0.3642 (2)	0.0768 (7)	
H9	0.8184	0.4218	0.3732	0.092*	
C10	0.9323 (4)	0.52263 (18)	0.3700 (2)	0.1001 (10)	
H10	0.8806	0.5639	0.3811	0.120*	
C11	1.0463 (4)	0.5467 (2)	0.3594 (2)	0.1051 (12)	
H11	1.0718	0.6038	0.3632	0.126*	
C12	1.1207 (3)	0.4857 (2)	0.3434 (2)	0.0959 (10)	
H12	1.1990	0.5015	0.3374	0.115*	
C13	1.0833 (2)	0.40133 (17)	0.33588 (16)	0.0702 (7)	
H13	1.1356	0.3605	0.3247	0.084*	
C14	0.67503 (19)	0.11513 (11)	0.24655 (15)	0.0468 (5)	
C15	0.6368 (3)	0.05200 (14)	0.28837 (19)	0.0765 (7)	
H15	0.6896	0.0452	0.3594	0.092*	
C16	0.5197 (4)	−0.00143 (18)	0.2246 (2)	0.1072 (11)	
H16	0.4934	−0.0446	0.2526	0.129*	
C17	0.4422 (3)	0.00873 (17)	0.1204 (2)	0.0877 (8)	
H17	0.3628	−0.0273	0.0776	0.105*	
C18	0.4805 (2)	0.07121 (15)	0.07889 (18)	0.0666 (6)	
H18	0.4269	0.0781	0.0078	0.080*	
C19	0.5977 (2)	0.12417 (13)	0.14139 (15)	0.0535 (5)	
H19	0.6250	0.1662	0.1127	0.064*	
C20	0.54307 (19)	0.28099 (11)	0.24114 (14)	0.0459 (5)	
C21	0.5506 (2)	0.26333 (14)	0.33038 (16)	0.0584 (5)	
H21	0.6423	0.2616	0.3931	0.070*	
C22	0.4205 (3)	0.24817 (15)	0.3260 (2)	0.0725 (6)	
H22	0.4244	0.2356	0.3858	0.087*	
C23	0.2860 (3)	0.25168 (17)	0.2335 (2)	0.0795 (7)	
H23	0.1988	0.2411	0.2308	0.095*	
C24	0.2785 (2)	0.27057 (17)	0.1450 (2)	0.0760 (7)	
H24	0.1864	0.2735	0.0826	0.091*	
C25	0.4079 (2)	0.28527 (14)	0.14842 (16)	0.0605 (6)	
H25	0.4035	0.2980	0.0884	0.073*	

### Atomic displacement parameters (Å^2^)

**Table:**

	*U*^11^	*U*^22^	*U*^33^	*U*^12^	*U*^13^	*U*^23^
O2	0.0549 (9)	0.0732 (11)	0.0938 (12)	0.0013 (8)	0.0430 (8)	−0.0117 (9)
O4	0.0486 (9)	0.0987 (13)	0.1444 (16)	−0.0064 (8)	0.0541 (10)	−0.0593 (12)
O7	0.1289 (16)	0.0814 (12)	0.1126 (14)	0.0391 (11)	0.0908 (13)	0.0146 (10)
O8	0.0851 (12)	0.0926 (14)	0.1211 (15)	0.0113 (10)	0.0675 (12)	0.0081 (11)
N1	0.0447 (9)	0.0748 (13)	0.0567 (10)	0.0156 (8)	0.0290 (8)	0.0017 (9)
N3	0.0438 (9)	0.0791 (14)	0.1104 (16)	−0.0068 (9)	0.0462 (10)	−0.0360 (11)
N5	0.0622 (11)	0.0797 (14)	0.0520 (11)	0.0232 (10)	0.0293 (9)	0.0050 (9)
C2	0.0385 (10)	0.0779 (16)	0.0564 (13)	0.0058 (10)	0.0224 (9)	−0.0146 (12)
C4	0.0348 (10)	0.0810 (17)	0.0710 (14)	0.0039 (10)	0.0206 (10)	−0.0295 (12)
C5	0.0441 (10)	0.0706 (15)	0.0443 (11)	0.0153 (10)	0.0196 (9)	−0.0054 (10)
C6	0.0468 (11)	0.0790 (16)	0.0500 (12)	0.0149 (10)	0.0270 (9)	0.0049 (11)
N6	0.0370 (8)	0.0558 (10)	0.0650 (10)	−0.0021 (7)	0.0260 (8)	−0.0019 (8)
N7	0.0370 (8)	0.0462 (9)	0.0559 (10)	0.0036 (7)	0.0182 (7)	0.0055 (7)
N8	0.0393 (8)	0.0468 (9)	0.0625 (10)	0.0031 (7)	0.0289 (7)	0.0130 (8)
C7	0.0377 (10)	0.0504 (12)	0.0524 (11)	−0.0005 (8)	0.0257 (8)	0.0006 (9)
C8	0.0458 (10)	0.0578 (13)	0.0475 (11)	−0.0093 (9)	0.0191 (9)	−0.0011 (9)
C9	0.0752 (15)	0.0629 (16)	0.0913 (18)	−0.0108 (12)	0.0452 (14)	−0.0139 (13)
C10	0.104 (2)	0.0608 (17)	0.101 (2)	−0.0072 (16)	0.0347 (18)	−0.0097 (15)
C11	0.105 (2)	0.070 (2)	0.0763 (19)	−0.0331 (18)	0.0098 (17)	0.0112 (15)
C12	0.0807 (19)	0.105 (2)	0.0703 (18)	−0.0443 (18)	0.0218 (15)	0.0132 (16)
C13	0.0525 (12)	0.0899 (18)	0.0590 (13)	−0.0203 (12)	0.0249 (10)	0.0025 (12)
C14	0.0392 (9)	0.0431 (11)	0.0566 (12)	0.0040 (8)	0.0256 (9)	0.0000 (9)
C15	0.0956 (18)	0.0578 (14)	0.0655 (15)	−0.0198 (13)	0.0378 (13)	0.0029 (11)
C16	0.137 (3)	0.081 (2)	0.092 (2)	−0.0555 (19)	0.057 (2)	−0.0047 (16)
C17	0.0882 (18)	0.0767 (18)	0.088 (2)	−0.0355 (14)	0.0420 (16)	−0.0208 (14)
C18	0.0613 (13)	0.0670 (15)	0.0630 (14)	−0.0035 (11)	0.0288 (11)	−0.0089 (11)
C19	0.0505 (11)	0.0527 (12)	0.0600 (13)	0.0007 (9)	0.0321 (10)	0.0009 (10)
C20	0.0417 (10)	0.0421 (11)	0.0577 (12)	0.0052 (8)	0.0299 (9)	0.0059 (9)
C21	0.0543 (12)	0.0613 (13)	0.0646 (13)	0.0073 (10)	0.0359 (11)	0.0101 (10)
C22	0.0781 (16)	0.0782 (16)	0.0897 (17)	0.0069 (13)	0.0643 (15)	0.0110 (13)
C23	0.0579 (14)	0.0906 (18)	0.112 (2)	−0.0022 (12)	0.0608 (16)	−0.0005 (15)
C24	0.0425 (11)	0.1013 (19)	0.0818 (17)	0.0049 (11)	0.0329 (11)	−0.0025 (14)
C25	0.0440 (11)	0.0753 (15)	0.0620 (13)	0.0082 (10)	0.0291 (10)	0.0074 (11)

### Geometric parameters (Å, °)

**Table:**

O2—C2	1.231 (3)	C11—C12	1.356 (4)
O4—C4	1.234 (2)	C11—H11	0.9300
O7—N5	1.224 (2)	C12—C13	1.369 (4)
O8—N5	1.233 (2)	C12—H12	0.9300
N1—C6	1.322 (3)	C13—H13	0.9300
N1—C2	1.362 (3)	C14—C15	1.367 (3)
N3—C4	1.371 (3)	C14—C19	1.375 (3)
N3—C2	1.379 (3)	C15—C16	1.376 (3)
N3—H3	0.8600	C15—H15	0.9300
N5—C5	1.422 (3)	C16—C17	1.364 (4)
C4—C5	1.434 (3)	C16—H16	0.9300
C5—C6	1.380 (3)	C17—C18	1.356 (3)
C6—H6	0.9300	C17—H17	0.9300
N6—C7	1.333 (2)	C18—C19	1.368 (3)
N6—C8	1.422 (2)	C18—H18	0.9300
N6—H6A	0.8600	C19—H19	0.9300
N7—C7	1.330 (2)	C20—C25	1.373 (3)
N7—C14	1.425 (2)	C20—C21	1.374 (3)
N7—H7	0.8600	C21—C22	1.383 (3)
N8—C7	1.337 (2)	C21—H21	0.9300
N8—C20	1.428 (2)	C22—C23	1.368 (3)
N8—H8	0.8600	C22—H22	0.9300
C8—C9	1.373 (3)	C23—C24	1.367 (3)
C8—C13	1.378 (3)	C23—H23	0.9300
C9—C10	1.383 (4)	C24—C25	1.382 (3)
C9—H9	0.9300	C24—H24	0.9300
C10—C11	1.375 (5)	C25—H25	0.9300
C10—H10	0.9300		
			
C6—N1—C2	117.20 (18)	C11—C12—C13	121.5 (3)
C4—N3—C2	127.8 (2)	C11—C12—H12	119.2
C4—N3—H3	116.1	C13—C12—H12	119.2
C2—N3—H3	116.1	C12—C13—C8	119.7 (3)
O7—N5—O8	121.2 (2)	C12—C13—H13	120.2
O7—N5—C5	119.5 (2)	C8—C13—H13	120.2
O8—N5—C5	119.33 (18)	C15—C14—C19	119.88 (18)
O2—C2—N1	122.85 (19)	C15—C14—N7	119.08 (18)
O2—C2—N3	119.2 (2)	C19—C14—N7	121.02 (17)
N1—C2—N3	117.9 (2)	C14—C15—C16	119.6 (2)
O4—C4—N3	119.4 (2)	C14—C15—H15	120.2
O4—C4—C5	129.0 (2)	C16—C15—H15	120.2
N3—C4—C5	111.65 (18)	C17—C16—C15	120.2 (2)
C6—C5—N5	118.7 (2)	C17—C16—H16	119.9
C6—C5—C4	119.1 (2)	C15—C16—H16	119.9
N5—C5—C4	122.10 (18)	C18—C17—C16	120.2 (2)
N1—C6—C5	126.0 (2)	C18—C17—H17	119.9
N1—C6—H6	117.0	C16—C17—H17	119.9
C5—C6—H6	117.0	C17—C18—C19	120.2 (2)
C7—N6—C8	127.70 (17)	C17—C18—H18	119.9
C7—N6—H6A	116.1	C19—C18—H18	119.9
C8—N6—H6A	116.1	C18—C19—C14	120.0 (2)
C7—N7—C14	124.30 (15)	C18—C19—H19	120.0
C7—N7—H7	117.8	C14—C19—H19	120.0
C14—N7—H7	117.8	C25—C20—C21	120.68 (18)
C7—N8—C20	124.48 (15)	C25—C20—N8	119.28 (18)
C7—N8—H8	117.8	C21—C20—N8	119.99 (17)
C20—N8—H8	117.8	C20—C21—C22	119.4 (2)
N7—C7—N6	118.00 (16)	C20—C21—H21	120.3
N7—C7—N8	121.24 (16)	C22—C21—H21	120.3
N6—C7—N8	120.75 (17)	C23—C22—C21	119.9 (2)
C9—C8—C13	119.7 (2)	C23—C22—H22	120.1
C9—C8—N6	123.18 (19)	C21—C22—H22	120.1
C13—C8—N6	117.1 (2)	C24—C23—C22	120.7 (2)
C8—C9—C10	119.4 (3)	C24—C23—H23	119.7
C8—C9—H9	120.3	C22—C23—H23	119.7
C10—C9—H9	120.3	C23—C24—C25	119.9 (2)
C11—C10—C9	120.7 (3)	C23—C24—H24	120.0
C11—C10—H10	119.6	C25—C24—H24	120.0
C9—C10—H10	119.6	C20—C25—C24	119.5 (2)
C12—C11—C10	118.9 (3)	C20—C25—H25	120.3
C12—C11—H11	120.5	C24—C25—H25	120.3
C10—C11—H11	120.5		
			
C6—N1—C2—O2	−176.90 (19)	C8—C9—C10—C11	−1.8 (4)
C6—N1—C2—N3	2.8 (3)	C9—C10—C11—C12	−0.1 (4)
C4—N3—C2—O2	173.4 (2)	C10—C11—C12—C13	1.1 (4)
C4—N3—C2—N1	−6.2 (3)	C11—C12—C13—C8	−0.2 (4)
C2—N3—C4—O4	−174.6 (2)	C9—C8—C13—C12	−1.7 (3)
C2—N3—C4—C5	5.8 (3)	N6—C8—C13—C12	−179.87 (19)
O7—N5—C5—C6	−169.02 (19)	C7—N7—C14—C15	−141.8 (2)
O8—N5—C5—C6	12.0 (3)	C7—N7—C14—C19	39.8 (3)
O7—N5—C5—C4	9.4 (3)	C19—C14—C15—C16	−0.8 (4)
O8—N5—C5—C4	−169.6 (2)	N7—C14—C15—C16	−179.2 (2)
O4—C4—C5—C6	178.0 (2)	C14—C15—C16—C17	−0.2 (5)
N3—C4—C5—C6	−2.4 (3)	C15—C16—C17—C18	0.4 (5)
O4—C4—C5—N5	−0.4 (3)	C16—C17—C18—C19	0.3 (4)
N3—C4—C5—N5	179.21 (18)	C17—C18—C19—C14	−1.3 (3)
C2—N1—C6—C5	0.1 (3)	C15—C14—C19—C18	1.5 (3)
N5—C5—C6—N1	178.24 (18)	N7—C14—C19—C18	179.85 (18)
C4—C5—C6—N1	−0.2 (3)	C7—N8—C20—C25	−136.65 (19)
C14—N7—C7—N6	−152.77 (18)	C7—N8—C20—C21	45.7 (3)
C14—N7—C7—N8	26.0 (3)	C25—C20—C21—C22	1.2 (3)
C8—N6—C7—N7	−168.86 (18)	N8—C20—C21—C22	178.88 (19)
C8—N6—C7—N8	12.4 (3)	C20—C21—C22—C23	−0.6 (4)
C20—N8—C7—N7	31.5 (3)	C21—C22—C23—C24	−0.4 (4)
C20—N8—C7—N6	−149.75 (18)	C22—C23—C24—C25	0.8 (4)
C7—N6—C8—C9	34.2 (3)	C21—C20—C25—C24	−0.8 (3)
C7—N6—C8—C13	−147.7 (2)	N8—C20—C25—C24	−178.5 (2)
C13—C8—C9—C10	2.7 (3)	C23—C24—C25—C20	−0.2 (4)
N6—C8—C9—C10	−179.3 (2)		

### Hydrogen-bond geometry (Å, °)

**Table:**

*D*—H···*A*	*D*—H	H···*A*	*D*···*A*	*D*—H···*A*
N6—H6A···O2^i^	0.86	1.94	2.794 (2)	174
N7—H7···N1^i^	0.86	2.21	2.934 (2)	142
N8—H8···O4^ii^	0.86	2.05	2.887 (2)	163

Symmetry codes: (i) −*x*+2, −*y*, −*z*+1; (ii) *x*, −*y*+1/2, *z*−1/2.
